# Influence of Clonidine and Ketamine on m-RNA Expression in a Model of Opioid-Induced Hyperalgesia in Mice

**DOI:** 10.1371/journal.pone.0079567

**Published:** 2013-11-01

**Authors:** Henning Ohnesorge, Zhiying Feng, Karina Zitta, Markus Steinfath, Martin Albrecht, Berthold Bein

**Affiliations:** 1 Department of Anesthesiology and Intensive Care Medicine, University Hospital Schleswig-Holstein, Campus Kiel, Kiel, Germany; 2 Department of Anesthesiology, the First Affiliated Hospital, Zhejiang University School of Medicine, Hangzhou, China; University of Würzburg, Germany

## Abstract

**Background:**

We investigated the influence of morphine and ketamine or clonidine in mice on the expression of genes that may mediate pronociceptive opioid effects.

**Material and Methods:**

C57BL/6 mice received morphine injections thrice daily using increasing doses (5-20mg∙kg^-1^) for 3 days (sub-acute, n=6) or 14 days (chronic, n=6) and additionally either s-ketamine (5mg∙kg^-1^, n=6) or clonidine (0.1mg∙kg^-1^, n=6). Tail flick test and the assessment of the mechanical withdrawal threshold of the hindpaw was performed during and 4 days after cessation of opioid treatment. Upon completion of the behavioural testing the mRNA-concentration of the NMDA receptor (NMDAR1) and β-arrestin 2 (Arrb2) were measured by PCR.

**Results:**

Chronic opioid treatment resulted in a delay of the tail flick latency with a rapid on- and offset. Simultaneously the mice developed a static mechanical hyperalgesia with a delayed onset that that outlasted the morphine treatment. Sub-acute morphine administration resulted in a decrease of NMDAR1 and Arrb2 whereas during longer opioid treatment the expression NMDAR1 and Arrb2 mRNA increased again to baseline values. Coadministration of s-ketamine or clonidine resulted in a reversal of the mechanical hyperalgesia and inhibited the normalization of NMDAR1 mRNA expression but had no effect on the expression of Arrb2 mRNA.

**Conclusion:**

In the model of chronic morphine therapy the antinociceptive effects of morphine are represented by the thermal analgesia while the proniceptive effects are represented by the mechanical hyperalgesia. The results indicate that the regulation of the expression of NMDAR1 and Arrb2 may be associated to the development of OIH in mice.

**Perspective:**

The results indicate that co-administration of clonidine or ketamine may influence the underlying mechanisms of OIH.

## Introduction

Opioids are a cornerstone of the treatment of moderate to severe pain, including acute pain, cancer-related pain, and chronic non-cancer pain [1]. Although common concerns regarding the use of opioids are the potential for detrimental side effects, physical dependence and addiction, accumulating evidence suggests that opioids may also cause another problem, often referred to as opioid-induced hyperalgesia (OIH) [2]. OIH is characterized by reduced nociceptive thresholds causing hyperalgesia and allodynia likely reflecting upregulation of compensatory pronociceptive pathways [2,3]. OIH has been shown in various clinical settings such as postoperative pain therapy [4] and short term opioid therapy in chronic pain patients [5]. Chronic opioid therapy results in changes of the sensory perception [6] and OIH may contribute to the clinical impression of opioid tolerance [7] resulting in a reduced pain intensity after opioid withdrawal in chronic pain patients [8]. The development of tolerance and OIH may be explained by the opponent-process theory [9,10] with opioid induced antinociceptive effects with a short on- and offset on one-hand and pronocieptive effects with a delayed on- and offset on the other hand.

Despite numerous investigations performed to elucidate OIH, the underlying mechanisms remain unclear. OIH is generally thought to result from the sensitization of pronociceptive pathways in the spinal cord and the brain. The most common hypothesis is that opioids may activate the N-Methyl-D-Aspartat (NMDA) receptor, mainly based on the observation that co-administration of ketamine in acute pain models abolished remifentanil-induced hyperalgesia [4,11,12]. Clinical and experimental observations also indicate that α2-adrenoceptor agonists may influence the development of OIH [3,12] as well as alleviate the symptoms of opioid-withdrawal [13]. The majority of studies carried out in exploring the mechanisms of OIH had focused on pathopysiological changes in the spinal cord. Since opioids also alter the pain processing in the brain, OIH may be associated with functional changes not only in the spinal cord. Possible factors in the development OIH may be the regulation of the expression of the NMDA-receptor as one main focus of pronociceptive effects of opioids and of the scaffolding protein β-arrestin 2 that is involved in the regulation of µ-opioid receptor internalization and re-expression [14]. 

The goals of this project were: 1) to establish a model of OIH in mice that reflects the clinical setting of chronic morphine therapy and to study the effects of opioid therapy on mechanical hyperalgesia and thermal analgesia; 2) to observe the effect of clonidine and ketamine in this model. 3) to measure the expression levels of β-arrestin 2 and NMDA receptor subunit 1 in the brain to determine further mechanisms of OIH.

## Materials and Methods

### Animals

All experiments were performed in accordance with the Guide for the Care and Use of Laboratory Animals from the National Academy of Science and the International Association for the Study of Pain guidelines on ethical standards for investigation in animals. The protocol was approved by the Committee on the Ethics of Animal Experiments of the University of Kiel and the Ministry of Energy, Agriculture, the **Environment** and Rural Areas, Schleswig-Holstein (Permit Number: V 312-72241.121-30 (61-5/06)). Adult male C57BL/6 mice weighing 20-30 g used for all experiments were bred in the animal facility of our institution. Mice were housed three per cage under a 12 hour dark/light cycle in a room with controlled temperature (22 ± 1 °C) and relative humidity (55 ± 10%). Food and water were available ad libitum except during behavioral evaluation. All experiments were conducted between 7:00 AM and 6:00 PM. During the study, animal welfare (feeding, posture, grooming, and motor activity) was verified daily.

### Drugs and experimental protocol

The clinically available injection form of the following drugs were used: morphine sulphate (Morphinsulfat-GRY^®^, Gry-Pharma GmbH, Kirchzarten, Germany), clonidine hydrochloride (Catapresan^®^, Boehringer Ingelheim, Ingelheim, Germany), and s-ketamine hydrochloride (Ketanest S^®^ , Pfizer, New York, USA). Morphine sulphate, clonidine hydrochloride and s-ketamine hydrochloride were dissolved and diluted in saline (0.9% NaCl) alone or combined, and were injected thrice daily (7:00 AM, 11:00 AM, and 4:00 PM) subcutaneously (s.c.) at the nape of the neck. These drugs were given in a total volume of 300 μL.

**Figure 1:**
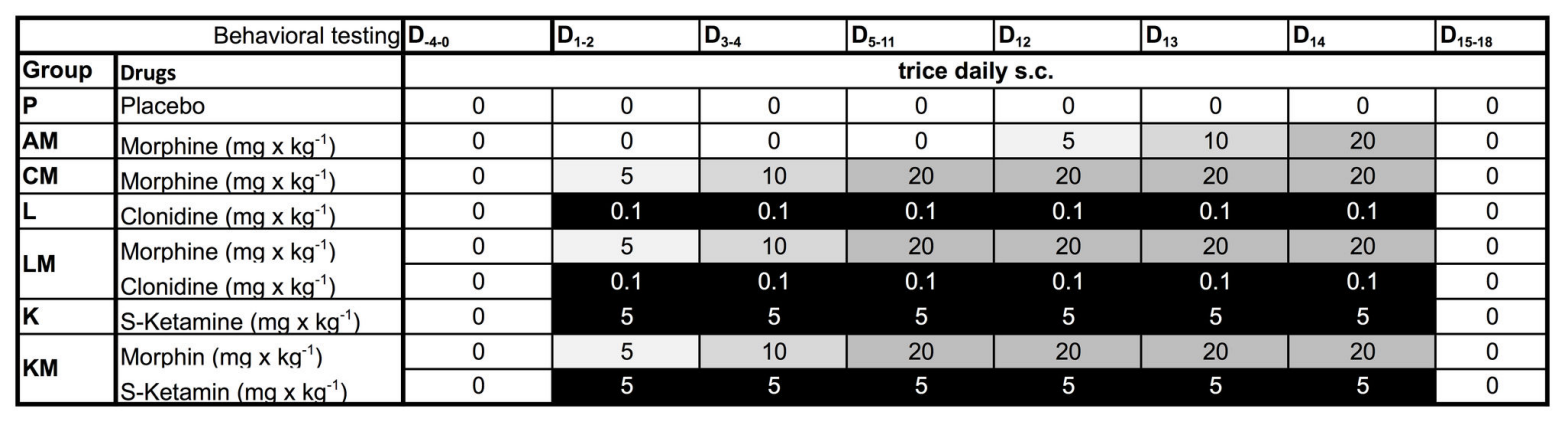
Protocol of drug administration. D: day. Numbers (0.1, 5, 10, 20) display the dose of drugs in mg·kg^-1^, 0 represents saline. Application was performed subcutaneously in a volume of 200-300µl thrice daily. N=6 animals per group.

The dosing scheme used for morphine administration is shown in Figure 1. Additionally to the treatment groups P (placebo), AM (sub-acute morphine) and CM (chronic morphine) four groups received morphine and either clonidine or s-ketamine. Group LM (clonidine/chronic morphine) received 0.1 mg/kg clonidine trice daily on D_1_-D_14_, group KM (ketamine/chronic morphine) 5mg/kg s-ketamine trice daily on D_1_-D_14_. Group L (clonidine/placebo) and group K (ketamine/placebo) received the same doses of clonidine and ketamine but without morphine on D_1_-D_14_. Six animals were assigned to each group. Four days after the last injection, mice were decapitated, brains were removed from the skull and immediately flash-frozen in liquid nitrogen and stored at -80 °C until RNA extraction.

### Behavioral testing

Before behavioral testing the animals were gently handled daily by the investigator, habituated to the experimental room environment and familiarized with the special conditions for evaluating pain behaviors for one week. Testing was performed in a quiet test room by the same investigator, who was blinded to the treatment of the mice. After the habituation period, baseline responses were obtained during 5 consecutive days for each paradigm in order to ensure stability (D_-4_, D_-3_, D_-2_, D_-1_ and D_0_). To exclude a time delay between the behavioral tests, we limited to number of mice tested on 3 per day. In all experiments, mechanical withdrawal thresholds of mice were measured daily in a period of 120–150 minutes after the first injection on each day (D_1_-D_14_). Tail flick latencies were determined immediately after the mechanical withdrawal thresholds 150-180 min after the first injection (D_1_-D_14_). 

#### Mechanical withdrawal thresholds

The withdrawal threshold on usually non noxious mechanical stimulation was assessed to determine a static mechanical hyperalgesia [15]. This paradigm was used because the reaction on non noxious mechanical stimulation is influenced not only by spinal but also in a greater extend by supraspinal mechanisms [16]. 

Static mechanical hyperalgesia was assessed by using a series of calibrated nylon von Frey filaments (Marstock Nerv test, Schriesheim, Germany), ranging from 0.16 g to 6 g with logarithmically incremental stiffness, according to the “up-down” algorithm described by Chaplan et al. [17]. In these experiments, mice were placed in clear plastic cages with a wire-mesh floor of 2.5×4 cm. Behavioral accommodation was allowed for approximately 30 minutes, until cage exploration and major rooming activities ceased. The von Frey filaments were applied to the plantar surface of the hind perpendicularly through the mesh floor with sufficient force to cause slight buckling against the paw, and held for approximately 10 seconds. Testing was initiated with the 1.0 mN filament, the strength of the next filament was decreased or increased according to the response, and the time interval between consecutive filament administrations was at least 5 seconds. If the mouse responded to touching the footpad with this filament by brisk withdrawal of the hindpaw, the response was considered as positive. In case of a positive response, the next weaker stimulus was presented; in case of a negative response, the next stiffer filament in the series was applied. This procedure was repeated until six responses either positive or negative were recorded. After 5 minutes test-free period, mechanical withdrawal threshold was measured again beginning with 1.0 mN until a brisk withdrawal of hindpaw was noted. This was repeated for three times. The smallest filament eliciting a foot withdrawal response from the three tests was considered to represent the withdrawal threshold. 

#### Tail flick latency test

The tail flick latency was assessed to determine the thermal analgesia. This paradigm was used because it reflects mainly a spinal reflex and is only under certain circumstances controlled by supraspinal mechanisms (for review see 16). 

Thermal analgesia was measured using a tail-flick unit (Ugo Basile, Comerio, Italy) [18]. Mice were immobilized except for free tail movement. Heat from an infrared source was administered to the tail with a radiation intensity of 40 mW×cm^-2^. Tail flick latency was defined as the time of heat exposure until withdrawal of the tail, which was recorded by a single blinded observer. In order to avoid tissue damage, a cut-off time of 10 seconds was defined as the maximum analgesic effect. The average tail flick latency time was obtained from three consecutive trials with an interval of about 5-10 minutes performed at three different sites of the tail.

### Molecular analysis of gene regulation in brain tissue

#### Brain tissue preparation

Mice were deeply anesthetized and decapitated 4 days after the last injection, brains were carefully removed, weighed, placed in individual tubes and immediately flash-frozen in liquid nitrogen. To minimize the risk of degradation of the mRNA the procedure was performed in less than 120 sec. after decapitation. Until RNA extraction was performed the brains were stored at -80 °C. 

#### RNA extraction

The frozen brain tissue was disrupted and homogenized by a rotor-stator homogenizer (Polytron®, PT 1200 E, Kinematica AG, Switzerland) for 90-120 seconds in a sterile glass tube containing 2 mL RLT buffer (Qiagen GmbH, Hilden, Germany) and 1% β-mercaptoethanol (Qiagen GmbH). Isolation of RNA was performed with the RNeasy minikit (Qiagen GmbH) according to the manufacturer’s protocol. The concentration and purity of the isolated RNA was measured using a spectrophotometer (Genesys TM spectrophotometer series 10, Rochester, N.Y., USA) at 260 nm and 280 nm, respectively and RNA samples were stored at −80 °C.

#### Reverse transcription (RT)

For reverse transcription of the isolated RNA the TaqMan® MicroRNA Reverse Transcription Kit (Applied Biosystems, California, USA) was used according to the manufacturer’s protocol. Employing this kit, cDNA was generated from 200 ng RNA using a Thermo cycler (Biometra® T1, Göttingen, Germany) and the following temperature profile: 10 minutes at 25 °C, 120 minutes at 37 °C, 5 seconds at 85 °C. The product was immediately chilled on ice and stored at -20 °C.

#### Polymerase chain reaction (PCR)

Primers were designed, synthesized (Metabion, Martinsried, Germany) and employed to amplify specific fragments of the mouse transcripts. The following primers were used: Arrestin beta 2 (*Arrb2*) (NM_145429.4): 5´-CTTCCCTGGACAAAGAGCTG-3´ and 5´-CAAGTGATGGGTCCAGTGTG-3´, annealing temperature 58 °C, amplicon size 760 bp; Glutamate receptor, ionotropic (*NMDAR1*) (NM_008169): 5´-GTCCTCTGCCATGTGGTTTT-3´ and 5´-CCAGATCGCACTTCTGTGAA-3´, annealing temperature 58 °C, amplicon size 431 bp; Hypoxanthine guanine phosphoribosyl transferase 1 (*Hprt1*) (NM_013556): 5´-TGCTCGAGATGTCATGAAGG-3´ and 5´-GCAAATCAAAAGTCTGGGGA-3´, annealing temperature 58 °C, amplicon size 690 bp; FBJ osteosarcoma oncogene (*Fos*) (NM_010234): 5´-ACTCCGGGCTGCACTACTTA-3´ and 5´-AATTGGAAC ACGCTATTGCC-3´, annealing temperature 58 °C, amplicon size 612 bp; Jun-B oncogene (*Junb*) (NM_008416): 5´-CCATCAGCTACCTCCCACAT-3´and 5´-TGCGTGTTTCTTCTCCACAG-3´, annealing temperature 58 °C, amplicon size 800 bp; arrestin beta 1 (*Arrb1*) (NM_178220): 5´-TGACACTGTAAGGGGCTGG-3´ and 5´-TGATCAGCAAAGCCACAAAG-3´, annealing temperature 58 °C, amplicon size 812 bp. Negative controls were performed by omitting the respective input cDNA. Each RT-PCR was repeated at least three times. All PCR experiments were performed employing Taq Polymerase from Solis BioDyne, Tartu, Estonia and a Biometra Thermo cycler (Biometra T1, Göttingen, Germany). PCR products were separated on 2.5% of agarose gels, followed by ethidiumbromide staining and were visualized by UV- transilumination. Images were taken and densitometrically analysed with the software ImageJ (v1.41o, NIH). Expression levels of the respective genes were calculated and displayed as the ratio of the intensities of the respective gene and *Hprt1*.

### Statistical analysis

To determine the baseline nociceptive threshold value, one-way analysis of variance (ANOVA) was performed to assess comparisons between the values obtained at D_-4_, D_-3_, D_-2_, D_-1_ and D_0_. When no statistically significant difference was found among D_-4_, D_-3_, D_-2_, D_-1_ and D_0_, the measurement of the nociceptive threshold performed on D_0_ was chosen as the baseline nociceptive threshold. Then the behavioral data were converted to the percentage of maximum possible effect (%MPE) for each animal at each day according to the following formula: %MPE = [(TL − BL)/ (cut-off value − BL)] × 100%, where BL was baseline threshold and TL was the value obtained during D_1_ -D_18_ [19,20]. The data are presented as means of %MPE ± SEM. To evaluate the time-course effects of treatments on the nociceptive threshold intra-group, one-way ANOVA followed by post hoc analysis using the Dunnett's test was performed for D_0_-D_18_. Between-group comparisons were performed using two-way ANOVA followed by Bonferroni’s multiple comparison tests. One-way ANOVA followed by post-hoc analysis using Dunnett's testing was performed for analysis of gene expression. Data are presented as mean ± SEM. A value of *P*<0.05 was considered statistically significant. All statistic analyses were performed using Prism 5.0 (GraphPad Software, Inc., San Diego, CA).

## Results

### Pain behavior

**Figure 2:**
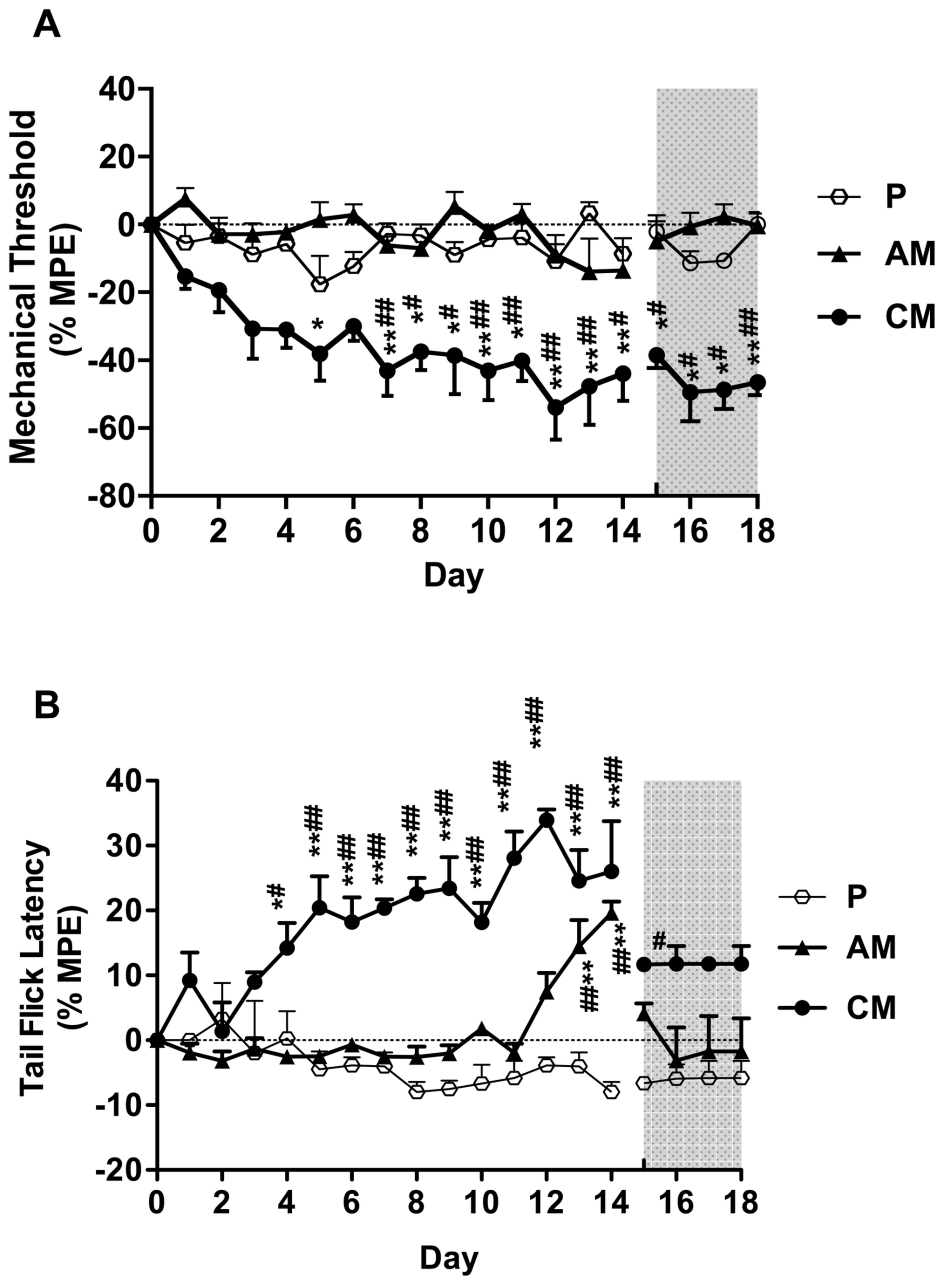
Effect of acute or chronic morphine administration on the mechanical threshold (A) and the tail flick latency (B). Mechanical threshold and tail flick latency were evaluated daily before injection (D_-4_-D_0,_ no significant changes compared to D_0_, data not shown), during injection (D_1_-D_14_) and after injection (D_15_-D_18_). The grey shaded surface represents 4 days after the final injection. The results are presented as % MPE ± SEM (n=6). P: placebo group; AM: acute morphine administration group; CM: chronic morphine administration group; *P<0.05; **P<0.01 compared with baseline; ^#^ *P*<0.05; ^##^ *P*<0.01 compared with group P.

**Figure 3:**
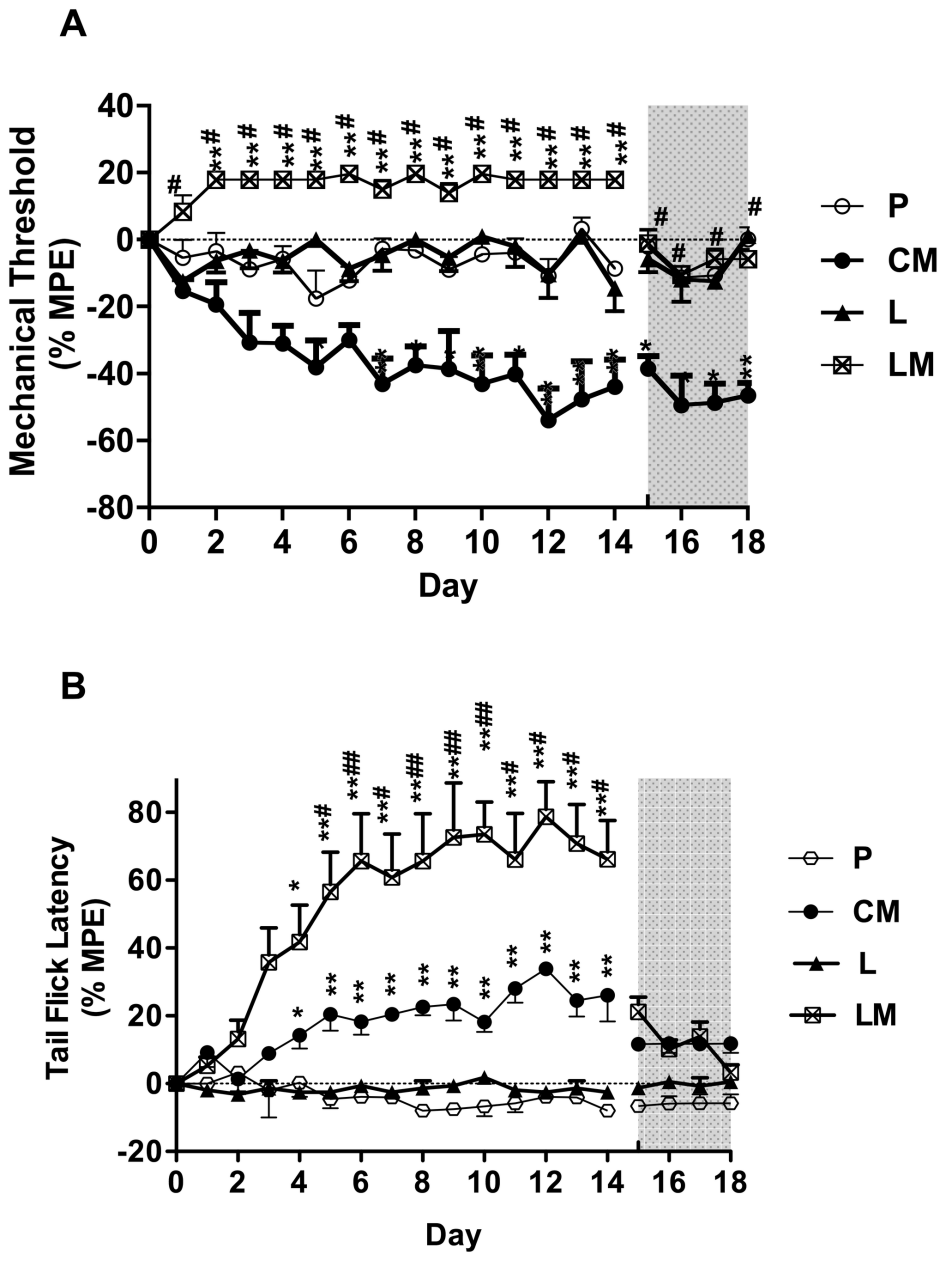
Effect of clonidine alone and in combination with chronic morphine administration on the mechanical threshold (A) and the tail flick latency (B). Mechanical threshold and tail flick latency were evaluated daily before injection (D_-4_-D_0_, no significant changes compared to D_0_, data not shown), during injection (D_1_-D_14_) and after injection (D_15_-D_18_). The grey shaded surface represents 4 days (D_15_-D_18_) after injection. The results are presented as %MPE ± SEM (n=6). P: placebo group; CM: chronic morphine administration group; L: clonidine administration group; LM: coadministration of morphine with clonidine group. *P<0.05, **P<0.01 compared with baseline; whereas ^#^ *P*<0.001 compared with group CM.

**Figure 4:**
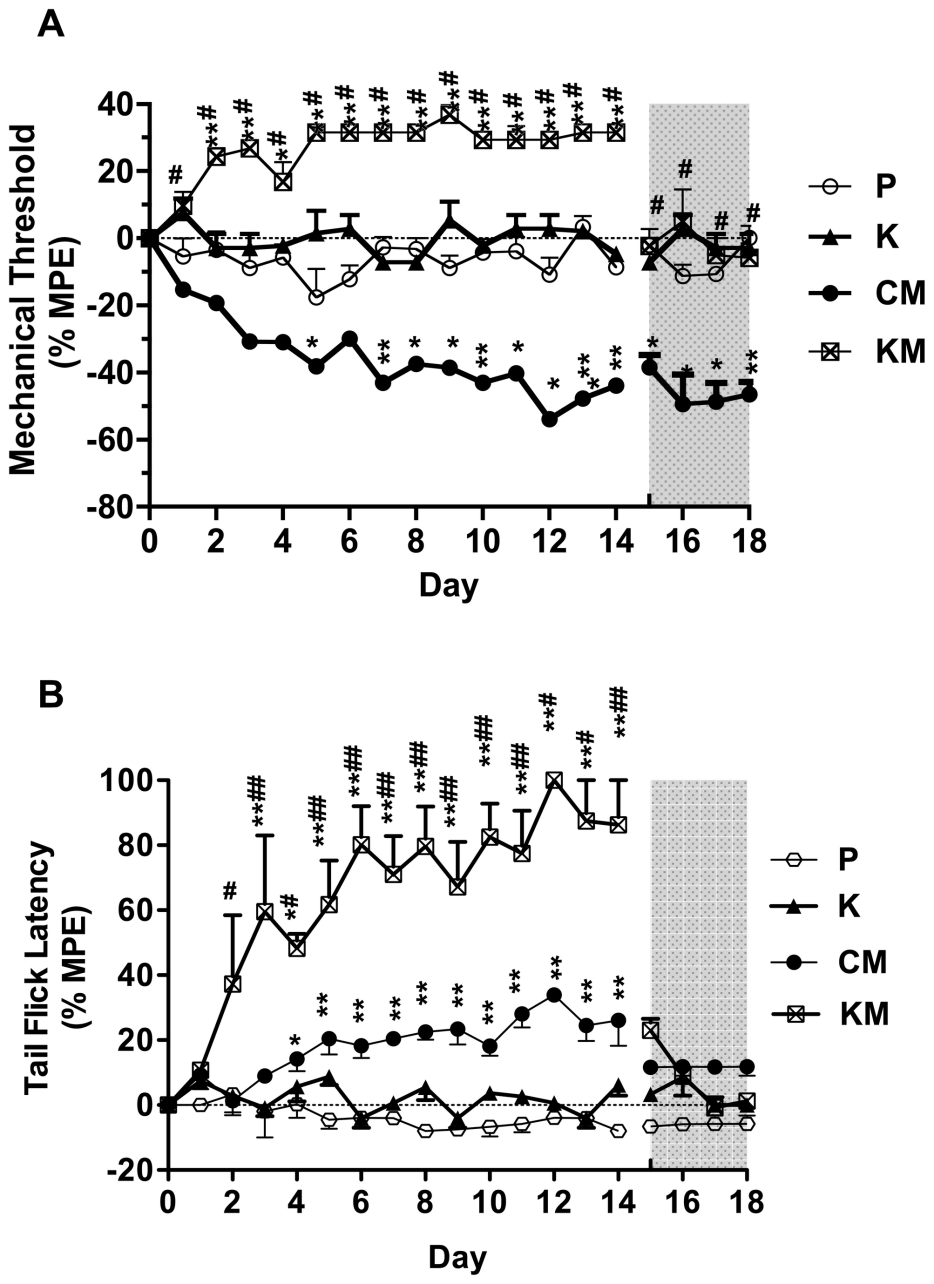
Effect of s-ketamine alone and in combination with chronic morphine administration on the mechanical threshold (A) and the tail flick latency (B). Mechanical threshold and tail flick latency were evaluated daily before injection (D_-4_-D_0_), during injection (D_1_-D_14_) and after injection (D_15_-D_18_). The grey shaded surface represents 4 days (D_15_-D_18_) after injection. The results are presented as %MPE ± SEM (n = 6). P: placebo group; CM: chronic morphine administration group; K: s-ketamine administration group; KM: coadministration of morphine with s-ketamine group. *P<0.05; **P<0.01 compared with baseline; whereas ^#^ *P*<0.01 compared with group CM.

No significant differences in baseline values of each group (D_-4_ to D_0_) or among the groups were found for both the mechanical thresholds and the tail flick latencies (*P*>0.05, data not shown). Short term morphine administration (group AM) resulted in no changes in mechanical hyperalgesia but in a delay of the tail flick latency during opioid-administration. Chronic opioid therapy (group CM) resulted in a decrease of the mechanical threshold that lasted at least 4 days longer than the morphine-administration, while the delay of the tail flick latency lasted not longer than the morphine treatment (Figure 2). Clonidine in a dose of 0.1 mg∙kg^-1^ (group L) did affect neither the mechanical threshold nor the tail flick latency. **Coadministration** of morphine and clonidine (group LM) resulted in an increase of the mechanical threshold that returned to baseline value immediately after end of treatment and a pronounced delay of the tail flick latency compared to group CM (Figure 3). Administration of **s-**ketamine in a dose of 5mg∙kg^-1^ (group K) resulted also in unchanged mechanical thresholds and tail flick latencies compared to group P. Compared to group CM, coadministration of s-ketamine and morphine (group KM) resulted in an increase of the mechanical threshold that returned to baseline value after end of treatment and a pronounced delay of the tail flick latency (Figure 4).

### Gene expression in brain tissue

**Figure 5:**
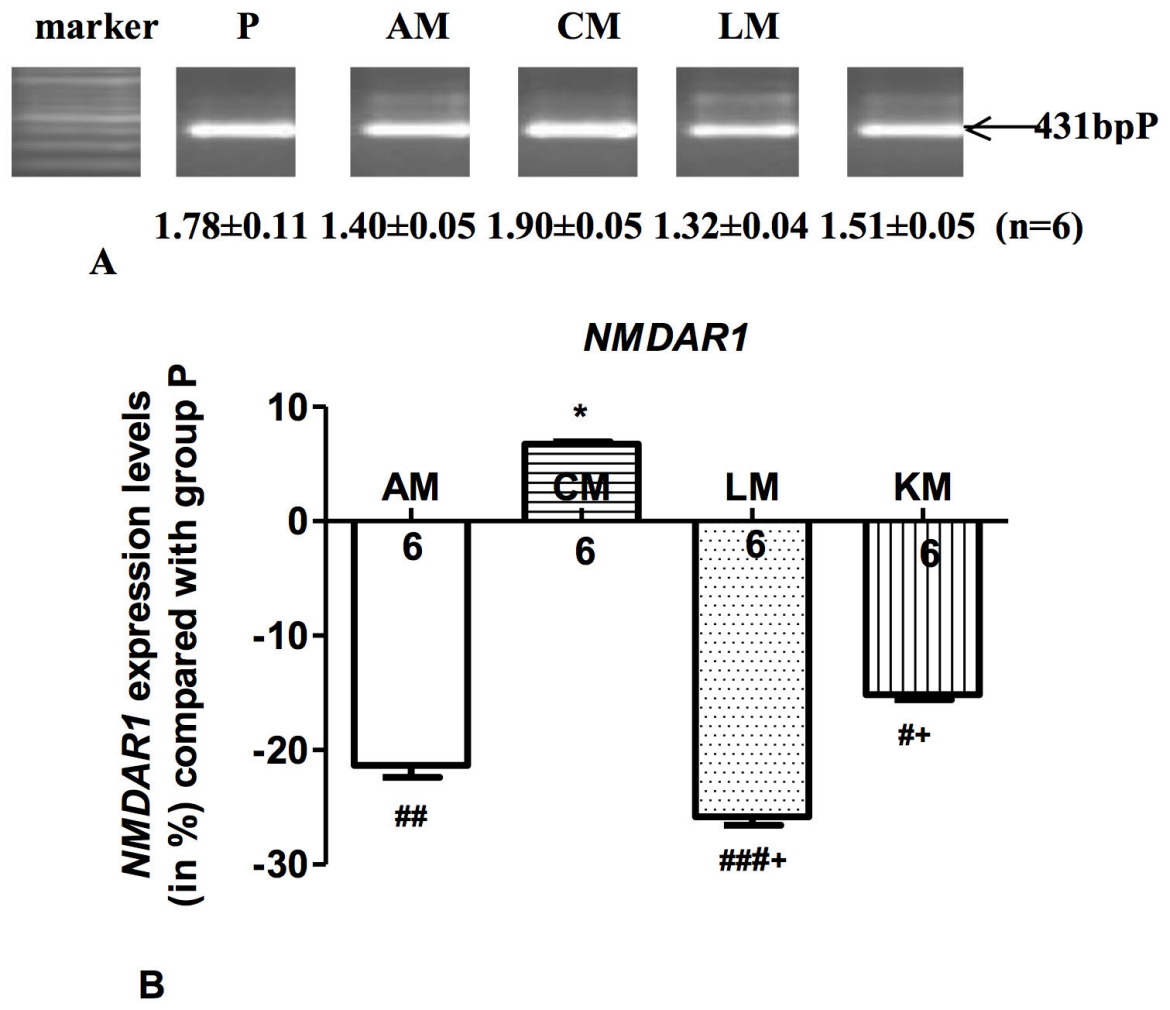
Regulation of *NMDAR1* mRNA in mice brain. Each PCR experiment was repeated 3 times per animal (n=6), one representative PCR result is shown (A). Values below the bands display the average level of band densities of *NMDAR1* compared with those of the housekeeping gene *Hprt1* (not shown) for each group (mean ± SEM). Regulation of *NMDAR1* mRNA expression in group AM, CM, LM and KM compared with group P (B). Columns show the mean upregulation or downregulation of *NMDAR1* mRNA expression in group AM, CM, LM and KM compared with group P, bars denote SEM; numbers display the numbers of mice in each group. AM: acute morphine administration group; CM: chronic morphine administration group; LM: coadministration of morphine with clonidine group; KM: coadministration of morphine with s-ketamine group. ^#^ *P*<0.05; ^##^ *P*<0.01; ^###^ *P*<0.001 compared with group P, *P<0.001 compared with group AM, and ^+^ *P*<0.001 compared with group CM.

**Figure 6:**
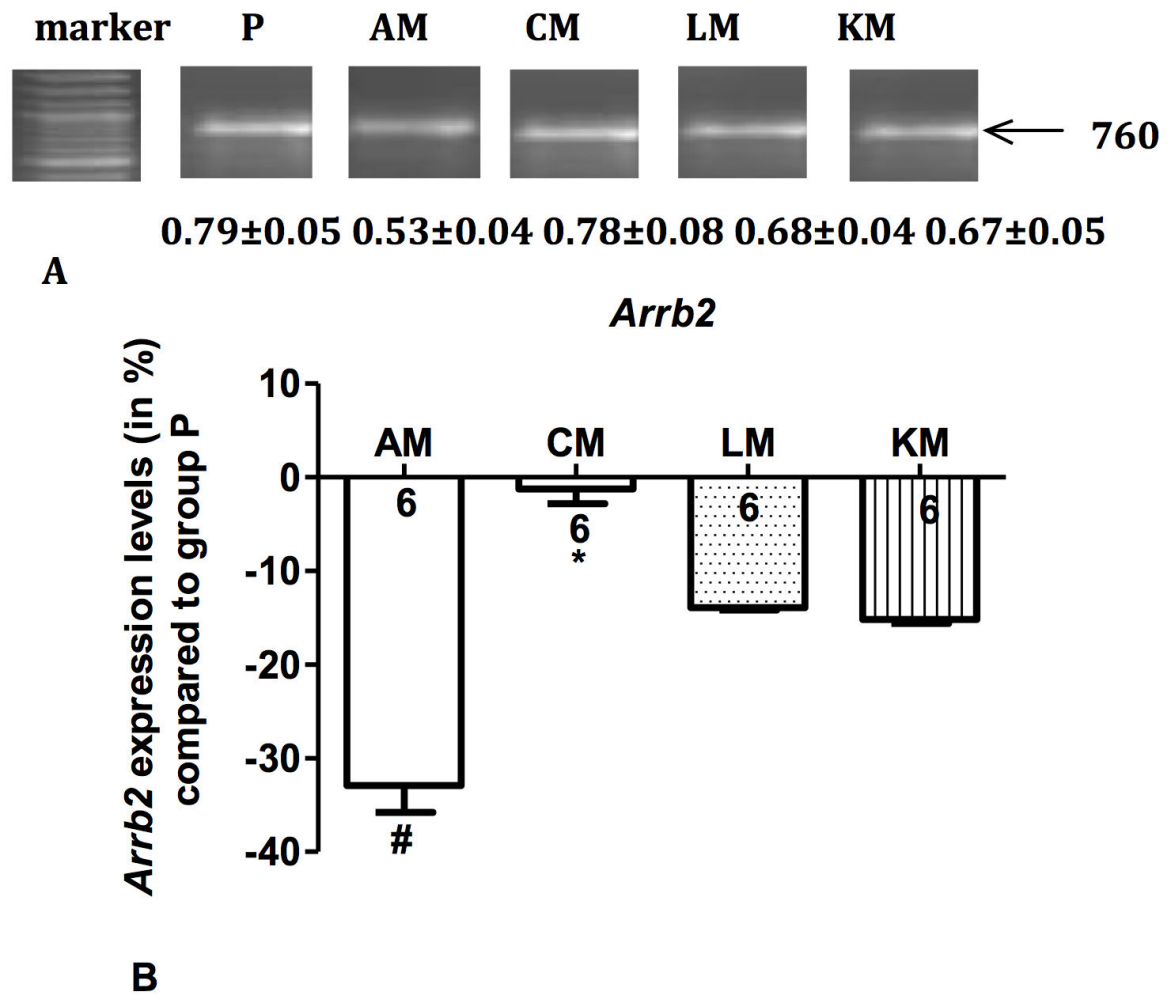
Regulation of β*-arrestin 2* (*Arrb2*) mRNA in mice brain. Each PCR experiment was repeated 3 times per animal (n=6), one representative PCR result is shown (A). Values below the bands display the average level of band densities of *Arrb2* compared with those of the housekeeping gene *Hprt1* (not shown) for each group (mean ± SEM). Regulation of *Arrb2* mRNA expression in group AM, CM, LM and KM compared with group P (B). Columns show the mean upregulation or downregulation of *Arrb2* mRNA expression in group AM, CM, LM and KM compared with group P, bars denote SEM; numbers display the number of mice in each group. AM: acute morphine administration group; CM: chronic morphine administration group; LM: coadministration of morphine with clonidine group; KM: coadministration of morphine with s-ketamine group. ^#^ *P*<0.01 compared with group P, *P<0.01 compared with group AM.

Compared with the placebo group, we observed a statistically significant downregulation both in *NMDAR1* mRNA expression and in *Arrb2* mRNA expression in group AM (Figures 5 and 6, *P*<0.01). After chronic morphine administration (group CM) gene expressions *of NMDAR1* mRNA and *Arrb2* mRNA were normalized to baseline value (*Arrb2, P*<0.01 vs. AM; *NMDAR1, P*<0.001 vs. AM) back to baseline values (p>0.05 compared to group P). Coadministration of s-ketamine (group KM) and clonidine (group LM) inhibited the normalization of the expression of *NMDAR1* mRNA (Figure 5), while both drugs had no effect on the expression of *Arrb2* (Figure 6). Neither sub-acute nor chronic morphine administration influenced the expression of c-Fos, Jun-b and Arrb1 mRNA. The expression of c-Fos, *Jun-b* and *Arrb1* mRNA was not influenced by the coadministration of clonidine or s-ketamine except of a significant upregulation of *Arrb*1 in group LM (p<0.05, data not shown).

## Discussion

The principal conclusions we draw from our data are:

1. A model of OIH in mice was established to simulate the clinical setting of an intermediate to long lasting opioid therapy. In this model tail flick latency test reflected the analgesic effect of opioids whereas the mechanical threshold reflected hyperalgesia as a main sign of a pronociceptive effect of opioids. This modification of the model initially introduced by Mao et al. [21] has the advantage of a missing or reduced development of tolerance for the tail flick latency and is fitting better to the opponent process theory than existing models of OIH.
2. Low dose s-ketamine and low dose clonidine treatment enhanced the analgesic effect and prevented the development of long-lasting hyperalgesia induced by morphine. S-ketamine and clonidine inhibited the normalization of *NMDAR1* mRNA expression during 14 days of opioid therapy, suggesting that regulation of NMDA receptors is involved in the mechanism of preventing OIH by s-ketamine or clonidine.

The first goal of our study was to establish a model of intermediate to long term opioid therapy in mice. As a modification of a model of opioid tolerance by intrathecal application of morphine published by Mao et al.[21], we used increasing systemically administered morphine doses to overcome the development of tolerance reflecting in an increased tail flick latency during the treatment period. Mao et al. determined a “narcotic induced thermal hyperalgesia” in the paw-withdrawal test as result of opioid tolerance. In the modified model we established we observed a static mechanical hyperalgesia during opioid treatment.

We suggest the increased delay of tail flick latency to reflect the analgesic property of opioid, and the development of mechanical hyperalgesia to reflect the pronociceptive effects. The time course of these effects with a short on- and offset of the analgesic effect and a delayed development of the hyperalgesic effect, that lasted for at least 4 days after termination of opioid treatment, matches to the opponent process theory [10] that was postulated by Laulin et al. for the effects of opioids [9]. The drug induced effects (such as opioid-induced analgesia) have a short onset and are stable upon repetition, whereas the counteracting process (such as opioid-induced hyperalgesia) has a delayed on- and offset, resulting clinically in a decreasing analgesia during treatment and a hyperalgesia after termination of treatment. Therefore, our model of OIH in C57BL/6 mice provides some new aspects of OIH. In our model signs of OIH developed are already detectable during opioid therapy. Thus this model may mirror the clinical observation that patients develop a paradoxal effect with an increased pain sensibility during chronic opioid therapy [4,5,7,8]. Also, the symptom of the pronociceptive effects of OIH – the mechanical hyperalgesia - can be distinguished from the analgesic effect of opioids measured by the tail flick latency.

Due to the fact that the tail flick latency represents a primarily spinal reflex with a simpler transduction of the signal we assume that OIH is result of more complex regulation mechanisms. This is consistent to results of Mao et al. [21] who used the heat induced tail flick latency to demonstrate only the opioid tolerance and the also heat-induced paw withdrawal test to determine the hyperalgesic effect of morphine, thus a modality specific effect by the used stimulus is excluded. Nevertheless a further evaluation of this model by additional behavioral tests is indicated to support this hypothesis.

In our model no hyperalgesic response was observed after sub-acute morphine treatment. This result seems to be inconsistent with a number of previous studies that indicate the development of hyperalgesia induced by morphine or other opioids after a single dose or several days of administration [22–26]. These studies usually investigated pain behavior hours to days after an acute administration of opioids, but not during the time window, where the analgetic effect was still present. Thus they determined hyperalgesia in opioid withdrawal not OIH during an ongoing opioid treatment. Using morphine in healthy mice Juni et al. [22] demonstrated that continuous infusion of both 8.0 and 40.0 mg/kg morphine resulted in an initial dose-dependent analgesic effect of about 2 days of duration followed by dose-dependent hyperalgesia starting on day 4. Thus a missing hyperalgesic effect in our model of sub-acute morphine treatment in healthy mice after 3 days of treatment is consistent with the results in a comparable model.

The coadministration of s-ketamine and clonidine resulted in a reversal of the mechanical hyperalgesia and in enhancement of the thermal analgesia in our model. Both drugs are known to attenuate the development of hyperalgesia in rodent models of OIH or neuropathic pain as well as in clinical settings of opioid therapy. Clonidine potentiates the antinociceptive effect of morphine [27–29], is used effectively in the management of opioid withdrawal [13] and attenuates hyperalgesia induced by opioids [12]. Ketamine reduces the hyperalgesic response on opioids but most studies investigated only the effect of pre-administration or co-administration of a single dose of NMDA receptor antagonists on OIH [7,12,23,30]. However, in our study, low dose s-ketamine was administrated chronically with morphine, reflecting the recommendations for the clinical use of ketamine [31]. This is the first time to demonstrate that chronic administration of low doses of clondine or s-ketamine reversed the hyperalgesia induced by morphine during and after morphine administration. The results can provide an additional explanation and experimental evidence for the beneficial effects of morphine combined with s-ketamine or clonidine for chronic pain patients [32,33].

Our study demonstrates further that opioid treatment has a biphasic effect on the expression of NMDAR1 mRNA in mice brain. Short term opioid administration resulted in a decreased expression of NMDAR1 mRNA whereas after cessation after longer opioid treatment the expression of NMDAR1 mRNA increased again to baseline values. This normalization is inhibited by coadministration of ketamine or clonidine suggesting that a downregulation of NMDAR1 mRNA is involved in the prevention of the development of OIH. NMDA receptor involvement in the mechanisms of OIH has also been shown elsewhere [12,22,23,34–39]., but our results suggests that regulation of *NMDAR1* mRNA expression in the brain may be involved in the mechanism of OIH. 

The results of our study also suggest that another underlying mechanism of OIH may be associated with the regulation of expression of β-arrestin 2 (*Arrb2*) mRNA. Compared with the placebo group, we observed a downregulation of *Arrb2* mRNA expression after sub-acute morphine treatment. Ongoing opioid treatment resulted in normalization to baseline value of gene expression of *Arrb2* mRNA. Arrb2 plays a critical role in internalization and re-expression of G-protein coupled receptors. *Arrb2* knockout mice exhibit an enhanced and prolonged antinociception of one dose of morphine followed by an ultimately developing tolerance, indicating that *Arrb2* is one important determinant of µ-opioid receptor desensitation and morphine tolerance [40,41]. This may be due to an Arrb2 dependent phosphorylation of the µ-opioid receptor [42]. Previous studies also demonstrated that chronic treatment with µ-opioid receptor (MOR) agonists resulted in a development of antinociceptive tolerance and caused a significant increase in expression of β-arrestin 2 in the cortex and striatum [43,44]. These data prompted us to hypothesize that downregulation of β-arrestin 2 expression in mice brain may lead to enhanced analgesia with primary morphine administration, whereas the normalization may refer to hyperalgesia during morphine long-term administration. This is supported by the trend that ketamine and clonidine inhibit this normalization during long term opioid treatment. Therefore, our results confirm previous studies, additionally demonstrating for the first time that OIH associated by a regulation of β-arrestin 2 mRNA. 

Our results indicate that Arrb1, Junb and c-Fos are not involved in the mechanism of opioid analgesia and hyperalgesia. Previous studies demonstrated that expression of these factors was altered after acute or chronic morphine administration [45]. This may be due to the fact that these genes are differentially regulated in different brain regions by morphine and we investigated the whole mice brain. Therefore, diluting and balancing effects cannot ruled out. This may reduce the relevance of the results of the regulation of NMDAR1 and Arrb2 mRNA expression. On the other hand, changes in mRNA expression of NMDAR1 and Arrb2 in brain areas responsible for pain perception may be underestimated by this method. This may explain the relative discrete changes of mRNA expression in our study. Nevertheless our results have to be confirmed on protein level in consideration of the regional distribution in the brain.

In our study we present a modified rodent model of OIH suitable for the investigation of mechanisms of pronociceptive effects of opioids. Further studies are needed to evaluate the functional relevance of the regulation of the changes in mRNA expression of NMDAR1 and Arrb2 during opioid treatment.

## References

[1] Boudreau D, Von Korff M, Rutter CM, Saunders K, Ray GT et al. (2009) Trends in long-term opioid therapy for chronic non-cancer pain. Pharmacoepidemiol Drug Saf 18: 1166-1175. doi:10.1002/pds.1833. PubMed: 19718704. 19718704 PMC3280087

[2] Angst MS, Clark JD (2006) Opioid-induced hyperalgesia: a qualitative systematic review. Anesthesiology 104: 570-587. doi:10.1097/00000542-200603000-00025. PubMed: 16508405. 16508405

[3] Chu LF, Angst MS, Clark DMDP (2008) Opioid-induced Hyperalgesia in Humans: Molecular Mechanisms and Clinical Considerations. Clin J Pain 24: 479-496. doi:10.1097/AJP.0b013e31816b2f43. PubMed: 18574358. 18574358

[4] Joly V, Richebe P, Guignard B, Fletcher D, Maurette P et al. (2005) Remifentanil-induced postoperative hyperalgesia and its prevention with small-dose ketamine. Anesthesiology 103: 147-155. doi:10.1097/00000542-200507000-00022. PubMed: 15983467. 15983467

[5] Chu LF, Clark DJ, Angst MS (2006) Opioid tolerance and hyperalgesia in chronic pain patients after one month of oral morphine therapy: a preliminary prospective study. J Pain 7: 43-48. doi:10.1016/j.jpain.2005.08.001. PubMed: 16414554. 16414554

[6] Chen L, Malarick C, Seefeld L, Wang S, Houghton M et al. (2009) Altered quantitative sensory testing outcome in subjects with opioid therapy. Pain 143: 65-70. doi:10.1016/j.pain.2009.01.022. PubMed: 19237249. 19237249 PMC2680088

[7] Mao J (2002) Opioid-induced abnormal pain sensitivity: implications in clinical opioid therapy. Pain 100: 213-217. doi:10.1016/S0304-3959(02)00422-0. PubMed: 12467992. 12467992

[8] Rome JD, Townsend CO, Bruce BK, Sletten CD, Luedtke CA et al. (2004) Chronic noncancer pain rehabilitation with opioid withdrawal: comparison of treatment outcomes based on opioid use status at admission. Mayo Clin Proc 79: 759-768. doi:10.1016/S0025-6196(11)62628-1. PubMed: 15182090. 15182090

[9] Laulin JP, Larcher A, Célèrier E, Le Moal M, Simonnet G (1998) Long-lasting increased pain sensitivity in rat following exposure to heroin for the first time. Eur J Neurosci 10: 782-785. PubMed: 9749743. 9749743 10.1046/j.1460-9568.1998.00083.x

[10] Solomon RL, Corbit JD (1974) An opponent-process theory of motivation. I. Temporal dynamics of affect. Psychol Rev 81: 119-145. doi:10.1037/h0036128. PubMed: 4817611. 4817611

[11] Angst MS, Koppert W, Pahl I, Clark DJ, Schmelz M (2003) Short-term infusion of the mu-opioid agonist remifentanil in humans causes hyperalgesia during withdrawal. Pain 106: 49-57. doi:10.1016/S0304-3959(03)00276-8. PubMed: 14581110. 14581110

[12] Koppert W, Sittl R, Scheuber K, Alsheimer M, Schmelz M et al. (2003) Differential modulation of remifentanil-induced analgesia and postinfusion hyperalgesia by S-ketamine and clonidine in humans. Anesthesiology 99: 152-159. doi:10.1097/00000542-200307000-00025. PubMed: 12826855. 12826855

[13] Gowing LR, Farrell M, Ali RL, White JM (2002) Alpha2-adrenergic agonists in opioid withdrawal. Addiction 97: 49-58. doi:10.1046/j.1360-0443.2002.00037.x. PubMed: 11895270. 11895270

[14] Koch T, Höllt V (2008) Role of receptor internalization in opioid tolerance and dependence. Pharmacol Therapeutics 117: 199-206. doi:10.1016/j.pharmthera.2007.10.003. PubMed: 18076994. 18076994

[15] Sandkühler J (2009) Models and mechanisms of hyperalgesia and allodynia. Physiol Rev 89: 707-758. doi:10.1152/physrev.00025.2008. PubMed: 19342617. 19342617

[16] Le Bars D, Gozariu M, Cadden SW (2001) Animal models of nociception. Pharmacol Rev 53: 597-652. PubMed: 11734620. 11734620

[17] Chaplan SR, Bach FW, Pogrel JW, Chung JM, Yaksh TL (1994) Quantitative assessment of tactile allodynia in the rat paw. J Neurosci Methods 53: 55-63. doi:10.1016/0165-0270(94)90144-9. PubMed: 7990513. 7990513

[18] Lichtman AH, Smith FL, Martin BR (1993) Evidence that the antinociceptive tail-flick response is produced independently from changes in either tail-skin temperature or core temperature. Pain 55: 283-295. doi:10.1016/0304-3959(93)90003-8. PubMed: 8121689. 8121689

[19] Osikowicz M, Mika J, Makuch W, Przewlocka B (2008) Glutamate receptor ligands attenuate allodynia and hyperalgesia and potentiate morphine effects in a mouse model of neuropathic pain. Pain 139: 117-126. doi:10.1016/j.pain.2008.03.017. PubMed: 18442882. 18442882

[20] Starowicz K, Bilecki W, Sieja A, Przewlocka B, Przewlocki R (2004) Melanocortin 4 receptor is expressed in the dorsal root ganglions and down-regulated in neuropathic rats. Neurosci Lett 358: 79-82. doi:10.1016/j.neulet.2003.12.096. PubMed: 15026153. 15026153

[21] Mao J, Price DD, Mayer DJ (1994) Thermal hyperalgesia in association with the development of morphine tolerance in rats: roles of excitatory amino acid receptors and protein kinase C. J Neurosci 14: 2301-2312. PubMed: 7908958. 7908958 10.1523/JNEUROSCI.14-04-02301.1994 PMC6577151

[22] Juni A, Klein G, Kest B (2006) Morphine hyperalgesia in mice is unrelated to opioid activity, analgesia, or tolerance: evidence for multiple diverse hyperalgesic systems. Brain Res 1070: 35-44. doi:10.1016/j.brainres.2005.11.054. PubMed: 16409995. 16409995

[23] Célèrier E, Rivat C, Jun Y, Laulin JP, Larcher A et al. (2000) Long-lasting hyperalgesia induced by fentanyl in rats: preventive effect of ketamine. Anesthesiology 92: 465-472. doi:10.1097/00000542-200002000-00029. PubMed: 10691234. 10691234

[24] Rivat C, Laulin JP, Corcuff JB, Célèrier E, Pain L et al. (2002) Fentanyl enhancement of carrageenan-induced long-lasting hyperalgesia in rats: prevention by the N-methyl-D-aspartate receptor antagonist ketamine. Anesthesiology 96: 381-391. doi:10.1097/00000542-200202000-00025. PubMed: 11818772. 11818772

[25] Waxman AR, Arout C, Caldwell M, Dahan A, Kest B (2009) Acute and chronic fentanyl administration causes hyperalgesia independently of opioid receptor activity in mice. Neurosci Lett 462: 68-72. doi:10.1016/j.neulet.2009.06.061. PubMed: 19559072. 19559072

[26] Célérier E, González JR, Maldonado R, Cabañero D, Puig MM (2006) Opioid-induced hyperalgesia in a murine model of postoperative pain: role of nitric oxide generated from the inducible nitric oxide synthase. Anesthesiology 104: 546-555. doi:10.1097/00000542-200603000-00023. PubMed: 16508403. 16508403

[27] Li X, Eisenach JC (2001) alpha2A-adrenoceptor stimulation reduces capsaicin-induced glutamate release from spinal cord synaptosomes. J Pharmacol Exp Ther 299: 939-944. PubMed: 11714880. 11714880

[28] De Kock M, Lavand'homme P, Waterloos H (2005) The short-lasting analgesia and long-term antihyperalgesic effect of intrathecal clonidine in patients undergoing colonic surgery. Anesth Analg 101: 566-572, table of contents doi:10.1213/01.ANE.0000157121.71808.04. PubMed: 16037177. 16037177

[29] Fairbanks CA, Wilcox GL (1999) Spinal antinociceptive synergism between morphine and clonidine persists in mice made acutely or chronically tolerant to morphine. J Pharmacol Exp Ther 288: 1107-1116. PubMed: 10027848. 10027848

[30] Li X, Angst MS, Clark JD (2001) A murine model of opioid-induced hyperalgesia. Mol Brain Res 86: 56-62. doi:10.1016/S0169-328X(00)00260-6. PubMed: 11165371. 11165371

[31] Himmelseher S, Durieux ME (2005) Ketamine for perioperative pain management. Anesthesiology 102: 211-220. doi:10.1097/00000542-200501000-00030. PubMed: 15618805. 15618805

[32] Koppert W, Schmelz M (2007) The impact of opioid-induced hyperalgesia for postoperative pain. Best Pract Res Clin Anaesthesiol 21: 65-83. doi:10.1016/j.bpa.2006.12.004. PubMed: 17489220. 17489220

[33] Chu LF, Angst MS, Clark D (2008) Opioid-induced hyperalgesia in humans: molecular mechanisms and clinical considerations. Clin J Pain 24: 479-496. doi:10.1097/AJP.0b013e31816b2f43. PubMed: 18574358. 18574358

[34] Crain SM, Shen KF (2000) Antagonists of excitatory opioid receptor functions enhance morphine's analgesic potency and attenuate opioid tolerance/dependence liability. Pain 84: 121-131. doi:10.1016/S0304-3959(99)00223-7. PubMed: 10666516. 10666516

[35] Li X, Angst MS, Clark JD (2001) A murine model of opioid-induced hyperalgesia. Brain Res. Mol Brain Res 86: 56-62. doi:10.1016/S0169-328X(00)00260-6. PubMed: 11165371. 11165371

[36] Mao J, Sung B, Ji RR, Lim G (2002) Chronic morphine induces downregulation of spinal glutamate transporters: implications in morphine tolerance and abnormal pain sensitivity. J Neurosci 22: 8312-8323. PubMed: 12223586. 12223586 10.1523/JNEUROSCI.22-18-08312.2002 PMC6758088

[37] Li X, Angst MS, Clark JD (2001) Opioid-induced hyperalgesia and incisional pain. Anesth Analg 93: 204-209. PubMed: 11429366. 11429366 10.1097/00000539-200107000-00040

[38] Célèrier E, Laulin J, Larcher A, Le Moal M, Simonnet G (1999) Evidence for opiate-activated NMDA processes masking opiate analgesia in rats. Brain Res 847: 18-25. doi:10.1016/S0006-8993(99)01998-8. PubMed: 10564731. 10564731

[39] DuPen A, Shen D, Ersek M (2007) Mechanisms of opioid-induced tolerance and hyperalgesia. Pain Manag Nurs 8: 113-121. doi:10.1016/j.pmn.2007.02.004. PubMed: 17723928. 17723928

[40] Bohn LM, Lefkowitz RJ, Caron MG (2002) Differential mechanisms of morphine antinociceptive tolerance revealed in (beta)arrestin-2 knock-out mice. J Neurosci 22: 10494-10500. PubMed: 12451149. 12451149 10.1523/JNEUROSCI.22-23-10494.2002 PMC6758751

[41] Bohn LM, Lefkowitz RJ, Gainetdinov RR, Peppel K, Caron MG et al. (1999) Enhanced morphine analgesia in mice lacking beta-arrestin 2. Science 286: 2495-2498. doi:10.1126/science.286.5449.2495. PubMed: 10617462. 10617462

[42] Appleyard SM, Celver J, Pineda V, Kovoor A, Wayman GA et al. (1999) Agonist-dependent desensitization of the kappa opioid receptor by G protein receptor kinase and beta-arrestin. J Biol Chem 274: 23802-23807. doi:10.1074/jbc.274.34.23802. PubMed: 10446141. 10446141

[43] Jiang B, Shi Y, Li H, Kang L, Ma L (2006) Decreased morphine analgesia in rat overexpressing beta-arrestin 2 at periaqueductal gray. Neurosci Lett 400: 150-153. doi:10.1016/j.neulet.2006.02.071. PubMed: 16563622. 16563622

[44] Hong D, Flood P, Diaz G (2008) The side effects of morphine and hydromorphone patient-controlled analgesia. Anesth Analg 107: 1384-1389. doi:10.1213/ane.0b013e3181823efb. PubMed: 18806056. 18806056

[45] Chang SL, Squinto SP, Harlan RE (1988) Morphine activation of c-fos expression in rat brain. Biochem Biophys Res Commun 157: 698-704. doi:10.1016/S0006-291X(88)80306-1. PubMed: 3144275. 3144275

